# SN38 PLGA nanoparticles provide sustained antitumor efficacy and ameliorate colitis severity in a murine colitis-associated cancer model

**DOI:** 10.1016/j.ijpx.2026.100552

**Published:** 2026-04-27

**Authors:** Meng-Tzu Weng, Shin-Yun Liu, Chia-Yueh Hsiung, Meng-Chuan Wu, Tsung-Ying Lee, Been-Ren Lin, Yunching Chen, Shu-Chen Wei

**Affiliations:** a Department of Internal Medicine, National Taiwan University Hospital and College of Medicine, Taipei, Taiwan; b Department of Medical Research, National Taiwan University Hospital, Hsin-Chu Branch, Hsinchu, Taiwan; c Liver Disease Prevention & Treatment Research Foundation, Taipei, Taiwan; d Institute of Biomedical Engineering, National Tsing Hua University, Hsinchu, Taiwan; e Institute of Biological Chemistry, Academia Sinica, Taipei, Taiwan; f Department of Surgery, National Taiwan University Hospital and College of Medicine, Taipei, Taiwan

**Keywords:** SN38, Poly (lactic-*co*-glycolic acid) (PLGA), Colorectal cancer, Colitis associated cancer

## Abstract

Irinotecan is a cornerstone therapy for metastatic colorectal cancer (CRC); however, the poor solubility and instability of its active metabolite, SN38, limit its therapeutic efficacy. This study aimed to improve SN38 delivery using poly(lactic-*co*-glycolic acid) (PLGA) nanoparticles and to evaluate their efficacy and safety in vitro and in a murine colitis-associated CRC model. SN38-PLGA nanoparticles exhibited a mean diameter of 170.7 ± 0.95 nm with a polydispersity index of 0.18 ± 0.04. In vitro, SN38-PLGA exhibited selective cytotoxicity—potent against HCT116 CRC cells but less toxic to HEK293T cells—and provided sustained SN38 release over 48 h. In the azoxymethane/dextran sodium sulfate–induced colitis-associated CRC model, intraperitoneal administration of either free SN38 or SN38-PLGA five times per week, combined with bevacizumab, significantly reduced tumor numbers compared with saline controls (7.2 vs. 7.7 vs. 14.8, *p* < 0.001), with comparable efficacy between the two treatment groups (*p* = 0.987). Notably, reducing SN38-PLGA dosing frequency from five to three times weekly maintained comparable efficacy (7.7 vs 5.5, *p* = 0.455). Moreover, SN38-PLGA markedly alleviated colitis severity, as demonstrated by improved body weight, increased colon length, reduced disease activity and histological scores, and lower serum levels of TNF-α, IL-1β, and IL-6 compared with free SN38 or saline. Histological evaluation revealed no significant damage to major organs, indicating a favorable safety profile. In conclusion, PLGA encapsulation preserves antitumor efficacy while reducing the severity of colitis. SN38-PLGA represents a promising therapeutic platform for colorectal cancer, with the potential to reduce dosing frequency and gastrointestinal toxicity.

## Highlights

• PLGA encapsulation prolongs SN38 release.
• PLGA-SN38 induces DNA damage with reduced cytotoxicity in non-cancerous cells.
• PLGA-SN38 achieves sustained antitumor efficacy with reduced dosing frequency in vivo.
• PLGA-SN38 alleviates colitis severity and preserves body weight in mice.

## Graphical abstract

**Figure:**
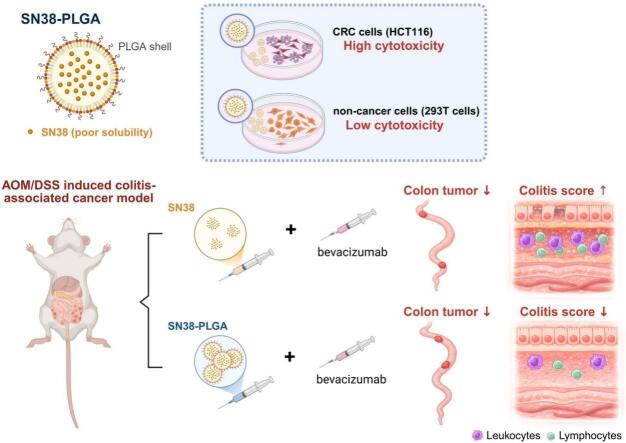

## 1 Introduction

Colorectal cancer (CRC) was the third most common cancer worldwide and the second leading cause of cancer-related deaths (Bray et al., 2024). The stage at diagnosis is the most critical factor for predicting survival. Patients with localized disease have a 5-year relative survival rate of 91%, whereas this rate drops dramatically to only 14% for those with distant metastases. Notably, 60% of new cases were diagnosed at an advanced stage, including 22% with distant metastases (Siegel et al., 2023). For patients with unresectable metastatic colorectal cancer (mCRC), systemic therapy is the cornerstone of first-line treatment. Standard regimens typically consist of combination chemotherapy, often enhanced by the addition of targeted agents such as anti-vascular endothelial growth factor (VEGF) or anti-epidermal growth factor receptor antibodies (Eng et al., 2024; Biller and Schrag, 2021). Bevacizumab, a monoclonal antibody targeting VEGF, has been shown to improve outcomes when combined with oxaliplatin-based regimens in both first- and second-line settings. It is also effective when combined with 5-fluorouracil alone or with irinotecan (Hurwitz et al., 2004). Despite advancements in systemic therapies, long-term survival remains poor, highlighting the critical need for new therapeutic approaches to improve patient outcomes.

Irinotecan, a topoisomerase I inhibitor, serves as a crucial chemotherapeutic agent in combination regimens for treating mCRC. Its active metabolite, 7-ethyl-10-hydroxycamptothecin (SN38), possesses 100–1000 times greater cytotoxic potency than irinotecan itself. Mechanistically, SN38 inhibits the religation of single-stranded DNA breaks (SSBs) generated during topoisomerase I activity. During DNA replication, these unrepaired SSBs cause replication fork collapse, resulting in DNA double-strand breaks (DSBs). The ensuing DNA damage activates the ATM and ATR signaling pathways, leading to Chk1 activation (phosphorylation) and cell cycle arrest. Irinotecan treatment also increases the expression of γ-H2AX, the phosphorylated form of the histone variant H2AX, which serves as a rapid and sensitive marker of DNA DSBs. γ-H2AX foci form at DSB sites to recruit DNA repair proteins, providing evidence of DNA damage and replication stress in tumor cells (Wang et al., 2018).

Despite its high potency, the clinical efficacy of SN38 is limited by pharmacokinetic and physicochemical barriers. Only 2–8% of administered irinotecan is converted to SN38 by carboxylesterase (Mathijssen et al., 2001), and SN38 is rapidly inactivated through glucuronidation catalyzed by uridine 5′-diphosphoglucuronosyltransferase 1A1 (UGT1A1). In addition, SN38 exhibits poor aqueous solubility and chemical instability—the active lactone form is readily hydrolyzed to an inactive carboxylate with a half-life of approximately 30 min at pH 7.3 (Palakurthi, 2015; Chourpa et al., 1998). Moreover, SN38 therapies are often accompanied by severe dose-limiting toxicities, particularly gastrointestinal side effects such as delayed-onset diarrhea (Xu et al., 2024), which further constrain its clinical application. Consequently, strategies that mitigate intestinal toxicity remain an important unmet clinical need.

To overcome the limitations of SN38's short half-life and intestinal toxicity, we developed a nanoparticle formulation by encapsulating SN38 within poly(lactic-*co*-glycolic acid) (PLGA). In contrast to conventional PLGA systems, this formulation incorporates lipid components and PEGylation (DSPE-PEG2000), along with D-α-tocopheryl polyethylene glycol succinate (TPGS) as a solubilizing and stabilizing agent, forming a hybrid polymer–lipid nanostructure (Gao et al., 2015). This design improves encapsulation stability, enhances colloidal stability, and enables controlled, sustained drug release. In addition, solvent displacement–driven self-assembly produces a reproducible nanostructure with consistent drug loading and release profiles. The sustained-release kinetics of the SN38-PLGA formulation may reduce dosing frequency, providing potential therapeutic advantages in inflammation-associated conditions. PLGA is biodegradable and biocompatible, undergoing hydrolysis into lactic acid and glycolic acid, which are subsequently metabolized and eliminated, thereby reducing systemic toxicity (Sadat Tabatabaei Mirakabad et al., 2014).

Therefore, the aim of this study was to evaluate the therapeutic efficacy and safety of SN38-loaded PLGA nanoparticles. We first conducted in vitro experiments using human colorectal cancer cells to evaluate the cytotoxic effects of SN38-loaded PLGA and confirm DNA damage induction. Subsequently, in vivo studies were performed to examine the efficacy and safety of combining SN38-loaded PLGA nanoparticles with anti-VEGF therapy in the azoxymethane/dextran sodium sulfate (AOM/DSS)-induced murine colorectal cancer model. This model enables the simultaneous evaluation of tumor suppression and intestinal inflammation, thereby providing a clinically relevant platform for therapeutic assessment.

## 2 Material and methods

### 2.1 Cell cultures

Human HEK293T cells and HCT116 colon cancer cells were purchased from ATCC and maintained in DMEM (Life Technologies) supplemented with 10% fetal bovine serum (FBS), l-glutamine, and 1% penicillin-streptomycin at 37 °C in a 5% CO2 incubator.

### 2.2 Preparation of PLGA-SN38

Briefly, the oil phase was prepared by mixing 211.5 μl DSPE-PEG2000 (2.8 kDa,10 mg/ml), 125 μl of PLGA (50:50, 17 kDa, 150 mg/ml), 125 μl of SN38 (30 mg/ml), 37.5 μl of DOPC (25 mg/ml), 46.88 μl of cholesterol (20 mg/ml), 93.75 μl TPGS (100 mg/ml) and 408 μl DMSO to reach a total volume of 1 ml. Subsequently, 40 μl of oil phase was added dropwise into 280 μl of deionized water under gentle stirring. The nanoparitlces (NPs) were self-assembled with continuous stirring for 30 min at room temperature. To collect NPs and remove organic solvent, the solution was centrifuged at 15000 rpm for 30 min at 4 °C. Finally, the obtained NPs were resuspended in a volume of water equal to the initial emulsion for further characterization.

Specifically, the nanoparticles are formed via a solvent displacement–driven self-assembly process, yielding a polymer–lipid hybrid core–shell architecture. During formulation development, we optimized the ratios and roles of each component to achieve stable nanostructures with controlled size, surface charge, and drug loading, consistent with prior reports demonstrating that TPGS, lipid content, and polymer composition critically regulate nanoparticle stability and assembly (Gao et al., 2015).

Mechanistically, PLGA forms the hydrophobic core encapsulating SN38, while TPGS enhances drug solubilization and stabilizes the core during nanoprecipitation. The lipid components (DOPC and cholesterol) assemble at the polymer–water interface to form a stabilizing layer that reduces water penetration and limits premature drug leakage, a feature previously shown to improve structural integrity and sustain drug release in lipid-coated PLGA systems. DSPE-PEG provides a hydrophilic corona that ensures colloidal stability and prolongs systemic circulation.

Overall, this hybrid architecture is the result of formulation optimization rather than simple mixing, enabling (i) efficient drug loading within the polymeric core, (ii) reduced burst release via the lipid interfacial barrier, and (iii) sustained release governed by PLGA degradation. These design principles are well supported by prior lipid-coated PLGA nanoparticle systems, which demonstrate enhanced structural stability, pharmacokinetics, and in vivo performance.

The SN38-loaded nanoparticles comprise a PLGA-based polymeric core encapsulating SN38 and are stabilized with lipid–PEG components, including DSPE-PEG, DOPC, cholesterol, and TPGS. For simplicity, this formulation is hereafter referred to as SN38–PLGA throughout the manuscript. For simplicity, this formulation is hereafter referred to as SN38–PLGA throughout the manuscript.

### 2.3 Dynamic light scattering analysis

Nanoparticles were prepared according to the method described in Section 2.2. Prior to measurement, the NP suspension was diluted 10-fold with deionized water. The hydrodynamic diameter and polydispersity index (PDI) were determined using a dynamic light scattering instrument (Zetasizer Pro, Malvern Panalytical, UK) at 25 °C.

### 2.4 Drug loading content and encapsulation efficiency

To determine the drug loading content (DLC) and encapsulation efficiency (EE), the NP pellets were collected via centrifugation and dissolved in DMSO. The concentration of SN38 was quantified using a UV/Vis spectrophotometer at 360 nm, based on the linear calibration curve established with known concentration of SN38 in DMSO.

The DLC was calculated using the following equation:

$$\mathrm{DLC}\;\left(\%\right)=\frac{\text{weight of encapsulated drug}\;\left(\mathrm{mg}\right)}{\text{weight of}\;\mathrm{SN}38-\text{PLGA}\;\left(\mathrm{mg}\right)}\times 100\%$$

The EE was calculated using the following equation:

$$\mathrm{EE}\;\left(\%\right)=\frac{\text{weight of encapsulated drug}\;\left(\mathrm{mg}\right)}{\text{weight of total drug added}}\times 100\%$$

### 2.5 In vitro drug release study

The release kinetics of SN38 from PLGA nanoparticles were evaluated in phosphate-buffered saline (PBS, pH 7.4) at 37 °C. The nanoparticle suspension (initially in deionized water) was divided into equal aliquots and transferred into individual microcentrifuge tubes. Each tube was then supplemented with 1 ml of PBS (pH 7.4), which served as the release medium. These tubes were then incubated on a shaking plate at 100 rpm and 37 °C. At predetermined time points, the tubes were centrifuged to pellet the nanoparticles. The resulting pellets were dissolved in DMSO, and the fluorescence intensity of SN38 was quantified using a multimode plate reader (Spark 10 M, Tecan, Germany) with excitation and emission wavelengths set at 355 nm and 575 nm, respectively. The SN38 signal at the initial time point (t_0_) was defined as 100%. The drug release percentage at any given time point (t_x_) was evaluated using the following equation:

$$\text{release}\;\left(\%\right)=\left(1-\frac{\text{signal}\;\mathrm{at}\;t_{x}}{\text{signal}\;\mathrm{at}\;t_{0}}\right)\times 100\%$$

### 2.6 Transmission electron microscopy

The morphology of the NPs was imaged using a high-contrast transmission electron microscope (HT7700, Hitachi, Japan). Prior to measurement, the NP suspension was diluted 200-fold with deionized water, and then 10 μl of the diluted sample was dropped onto a carbon-coated copper grid. The samples were then vacuum-dried overnight at room temperature to ensure complete dehydration. Imaging was performed at an accelerating voltage of 100 kV in high-contrast mode to observe the size and structure of the NPs.

### 2.7 Stability study of SN38-PLGA nanoparticles

To ensure the stability of SN38-PLGA NPs throughout the 4-week administration period, the NP suspension were lyophilized into a dry powder for long-term storage and resuspended in deionized water before each use. To verify the stability and integrity of the NPs after the lyophilization, we evaluated both the EE and size of nanoparticles. To assess whether the lyophilization process caused drug leakage, we compared the EE of fresh and lyophilized NPs. For freshly prepared NPs, the nanoparticle suspension was centrifuged and the pellet was dissolved in DMSO for quantification. For lyophilized NPs, the suspension was lyophilized for 24 h. The resulting powder was resuspended in deionized water and centrifuged to remove any leaked drug, and the collected pellet was dissolved in DMSO for quantification. The particle sizes of both freshly prepared and lyophilized nanoparticles were measured as described in Section 2.3.

### 2.8 Western blotting

HCT116 cells were seeded in 6-well plates (2.5 × 10^5^ cells/well) and incubated for 24 h prior to treatment with SN-38 or SN38–PLGA for 4 or 24 h. After treatment, cells were washed three times with ice-cold PBS and lysed using RIPA buffer (Cell Signaling Technology, MA, USA) supplemented with cOmplete™ Ultra protease inhibitor and PhosSTOP™ phosphatase inhibitor cocktails (Roche, Mannheim, Germany). The lysates were briefly sonicated and then centrifuged at 15,000 rpm at 4 °C for 20 min. Total protein concentration was determined using the Bradford Protein Assay (Bio-Rad, CA, USA). Equal amounts of protein (50 μg per lane) were mixed with 5 × SDS loading buffer, denatured at 95 °C for 5 min, and resolved by 10% SDS-PAGE. Proteins were transferred to PVDF membranes (Cytiva, MA, USA), which were then blocked with FastBlocking 1-min Protein-Free Buffer (BIOMAN, Taipei, Taiwan) for 1 min at room temperature. Membranes were incubated overnight at 4 °C with the following primary antibodies (all diluted 1:1000): phospho-CHK1 (Ser345, #2348), H2AX (#7631), γ-H2AX (Ser139, #9718), and Vinculin (#13901) from Cell Signaling Technology (MA, USA); and CHK1 (sc-8408) from Santa Cruz Biotechnology (CA, USA). After washing, membranes were incubated with HRP-conjugated anti-rabbit (#111–035-144, 1:10,000) or anti-mouse (#115–035-146, 1:10,000) secondary antibodies (Jackson ImmunoResearch, PA, USA) for 1 h at room temperature. Protein bands were visualized using the WestEZ ECL HRP UltimaPlus kit (BIOMAN, Taiwan) and captured with the FluorChem HD2 Imaging System (Alpha Innotech). Vinculin was used as an internal loading control to normalize protein levels. Densitometric analysis was performed using ImageJ software (NIH, USA) to quantify protein expression levels.

### 2.9 MTT assay

Cell viability was assessed using the MTT assay [3-(4,5-dimethylthiazol-2-yl)-2,5-diphenyltetrazolium bromide] (Sigma-Aldrich). Human colon cancer (HCT-116) and non-cancerous (HK293T) cells were seeded in 96-well plates (*n* = 3) at a density of 1 × 10^6^ cells per well. After 24 h, the medium was replaced with 200 μL of fresh DMEM containing increasing concentrations of SN38 or SN38-loaded composites. Cells were then incubated at 37 °C for 24 or 48 h. At the end of each incubation period, 20 μL of MTT solution was added to each well and incubated for an additional 4 h at 37 °C. Subsequently, the medium was removed, and 200 μL of DMSO was added, followed by shaking at 50 rpm for 20 min. Finally, the absorbance was measured at 450 nm using a microplate reader (Bio-Rad Laboratories Inc., Hercules, CA, USA). The final results were expressed as the percentage of viable cells compared with that of untreated control cells.

### 2.10 Animals

WT mice (BALB/c strains), aged 7–10 weeks, were obtained from the National Laboratory Animal Center (NLAC, NARLabs, Taipei, Taiwan). All animals were raised in temperature-controlled rooms (23 ± 2 °C) with 12 h light–dark cycles. Experimental procedures were approved by Ethics Committee for Animal Care and Use of the National Taiwan University College of Medicine (IACUC no. 20210085) and conducted in compliance with the institutional animal research guidelines and reported in accordance with the ARRIVE 2.0 guidelines.

### 2.11 SN38 or SN38-PLGA combined with Bevacizumab in AOM/DSS induced colitis-associated cancer models

**Fig. 1:**
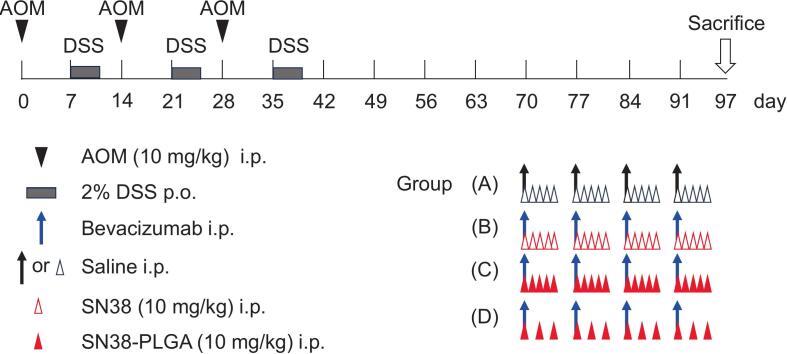
Schematic timeline illustrating the treatment schedule for saline, SN38 combined with bevacizumab, and SN38-PLGA combined with bevacizumab in the AOM/DSS induced colorectal cancer model. The mice were allocated into four groups: (1) saline group; (2) Bevacizumab combined with SN38 administered five times per week; (3) Bevacizumab combined with SN38-PLGA administered five times per week (4) Bevacizumab combined with SN38-PLGA administered three times per week. i.p. = intraperitoneal injection. p.o. = per oral (oral administration).

The mice were administered an intraperitoneal injection of 10 mg/kg AOM (Sigma, St. Louis, MO, USA) on day 0 of the experiment. Seven days later, 2% DSS (MP Biomedicals, Santa Ana, CA, USA) was provided in the drinking water for four days, followed by three days 14of regular water. This AOM/DSS cycle was repeated three times. Following completion of the three induction cycles, mice were randomly allocated into four treatment groups (*n* = 10 per group): (1) saline, (2) SN38 administered intraperitoneally five times per week plus bevacizumab, (3) SN38-PLGA administered intraperitoneally five times per week plus bevacizumab, and (4) SN38-PLGA administered intraperitoneally three times per week plus bevacizumab. Therapeutic intervention was initiated on day 69 after the start of the experiment. On day 97, mice were euthanized, and colon length, tumor count, and tumor area were assessed (Fig. 1). Bevacizumab (Avastin, Genentech, CA, USA) was administered intraperitoneally at a dose of 5 mg/kg once weekly. SN38 (10 mg/kg, based on SN38 content), SN38-PLGA (equivalent to 10 mg/kg SN38), or saline was administered intraperitoneally according to the designated schedule over a four-week period. The free SN38 dose was selected as 10 mg/kg according to previously published studies (Zhu et al., 2024; Zhu et al., 2025) and our preliminary dose-finding experiments. The administered dose in the SN38-PLGA was calculated based on the equivalent amount of active SN38. We administered the anti-tumor medication via intraperitoneal (i.p.) injection for practical and pharmacological reasons. Repeated tail-vein injections in longitudinal studies risk vascular injury, tail necrosis, and animal stress, whereas i.p. dosing ensures greater consistency, reproducibility, and welfare. Additionally, i.p. delivery in murine CRC models creates a peritoneal-to-plasma concentration gradient, enhancing local exposure to intestinal tumors before systemic distribution and improving therapeutic efficacy.

Following euthanasia, the colon was excised and gently flushed with ice-cold PBS to remove residual fecal material. It was then opened longitudinally from the cecum to the rectum and flattened with the mucosal surface facing upward against a green background. The tissue was fixed in 4% paraformaldehyde. Tumor quantification was conducted using ImageJ software (version 1.54p; National Institutes of Health, NIH, USA). Each image was calibrated according to the embedded scale bar. Macroscopically visible tumors were identified, individually outlined using the freehand selection tool, and their areas were recorded. Tumor number was defined as the total count of distinct lesions across the entire colon of each experimental mouse. All image assessments were performed in a blinded manner to reduce observer bias.

### 2.12 Disease activity index

A disease activity index (DAI) score was measured by combining the scores of weight loss (WL) (>5% WL = 0; 6–10% WL = 1; 11–15% WL = 2), fecal consistency (pellet = 0; pasty = 2; liquid = 4), and blood in stools (assessed with hemoccult kit negative = 0, positive = 2, and gross bleeding = 4) (Bader et al., 2018). The body weight and DAI were assessed every week since day 70.

### 2.13 Colitis score

The colitis scores were determined according to previously established criteria (Karmele et al., 2019). A blinded pathologist assessed tissue sections for goblet cell depletion, leukocyte infiltration, and submucosal inflammation, assigning scores on a scale from 0 to 3, where 0 indicates no pathology and 3 represents the most severe pathology. The three equally weighted subscores were then summed to generate a total histological colitis severity score ranging from 0 to 9.

### 2.14 Hematoxylin and eosin stain

Tumor tissues, heart, liver, kidney, lung and spleen from different groups (PBS, free SN38, and SN38 encapsulated PLGA) were fixed in 10% formalin and embedded in paraffin. Then, paraffin sections were cut to a thickness of 5 μm, stained with H&E, and observed by a pathologist under an optical microscope.

### 2.15 Quantification of serum cytokines by ELISA

Serum concentrations of TNF-α, IL-1β, and IL-6 were quantified using mouse-specific sandwich ELISA kits (Elabscience; TNF-α: *E*-EL-M3063, IL-1β: E-EL-M0037, IL-6: E-EL-M0044) according to the manufacturer's protocols. Whole blood was collected from experimental mice and centrifuged at 1000 ×*g* for 20 min at 4 °C. The supernatant was harvested and stored at −80 °C until analysis.

For cytokine measurement, serum samples and serially diluted standards were added to microplate wells pre-coated with capture antibodies specific for each cytokine. Following incubation, biotinylated detection antibodies were applied, and unbound materials were removed through repeated washing steps. HRP-conjugated streptavidin was then added, followed by incubation and subsequent washes. Tetramethylbenzidine substrate was introduced to initiate color development, and the reaction was terminated using stop solution. Optical density was measured at 450 nm using a multifunctional microplate reader (Hidex Sense).

### 2.16 Statistical analysis

All data were expressed as mean ± S.E.M. Comparisons between each group were analyzed by student *t-*test or one-way ANOVA followed by the Bonferroni test post-hoc test for multiple comparisons. Differences were considered statistically significant at * *p* < 0.05, **p < 0.01 and ***p < 0.001.

## 3 Results

### 3.1 Characterization of SN38-PLGA nanocomposites

**Fig. 2:**
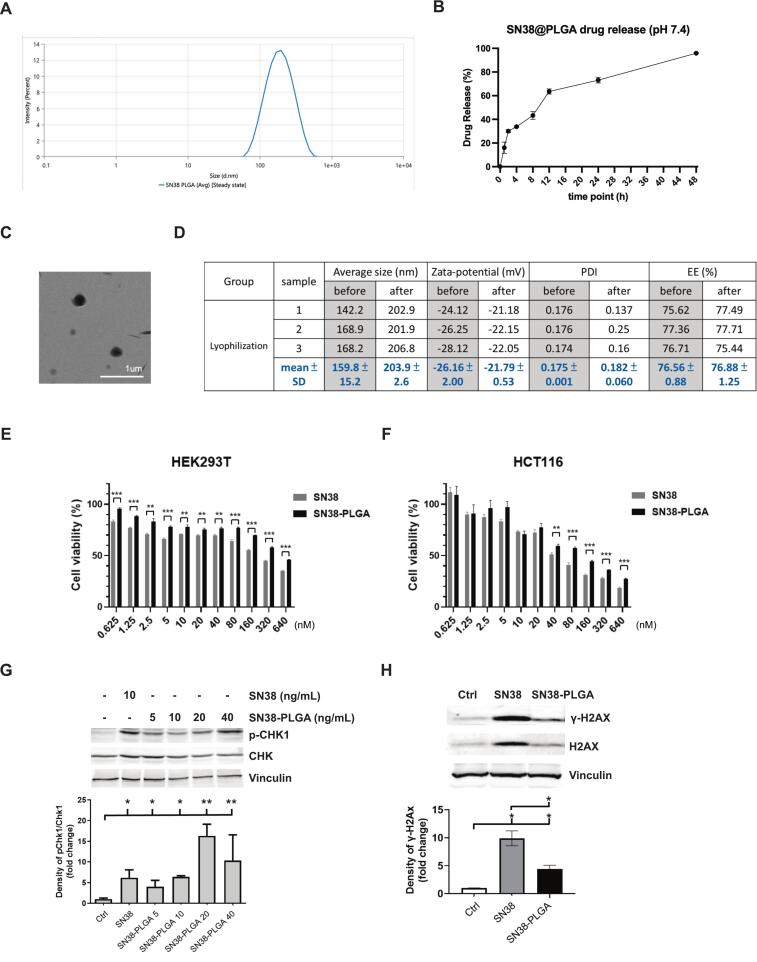
(A) Particle size distributions and polydispersity index (PDI) of the SN38-PLGA nanoparticles. (B) Accumulative drug release profile of SN38-PLGA at 7.4. (C) TEM images of PLGA-SN38. (D) The size, zeta potential, PDI and encapsulation efficiency (EE) % of SN38-PLGA before and after the lyophilization. (E) Cell viability of SN38 and SN38-PLGA was assessed in HEK293T cells after 48 h. (F) Cell viability of SN38 and SN38-PLGA was evaluated in HCT116 colorectal cancer cells after 48 h. Western blot results demonstrating the expression of (G) phospho-Chk1 in HCT116 cells following 4-h treatment with SN38 or SN38-PLGA and (H) γ-H2Ax in HCT116 cells following 24-h treatment with SN38 (20 nM) or SN38-PLGA (20 nM). Representative blots are shown in the upper panels, and quantitative data are presented in the lower panels. * *p* < 0.05, **p < 0.01 and ***p < 0.001.

The SN38-encapsulated PLGA nanoparticles exhibited an average diameter of 170.7 ± 0.95 nm with a PDI of 0.18 ± 0.04 and a zeta potential of −27.91 ± 1.05 mV, as measured by dynamic light scattering (Fig. 2A). The DLC and EE of SN38 within SN38-PLGA nanoparticles were approximately 8.55 ± 0.5% (wt/wt) and 81.73 ± 4.76%, respectively. The in vitro release profile of SN38 from PLGA nanoparticles was evaluated under physiological condition (pH 7.4, 37 °C). As shown in Fig. 2B, SN38 exhibited a sustained release pattern, with approximately 60% of the drug released within the first 12 h, reaching nearly 95% by 48 h. The transmission electron microscope (TEM) images of SN38-PLGA nanoparticles revealed well-dispersed, spherical particles with smooth surfaces, as shown in Fig. 2C. To assess the stability and structural integrity of the NPs following lyophilization and subsequent reconstitution, we performed a comprehensive physicochemical evaluation. The average diameter of the reconstituted NPs was 203.9 nm, representing a modest increase compared with freshly prepared NPs (159.8 nm). This size remains within the optimal range for passive targeting and systemic administration. Such an increase is commonly observed after lyophilization and rehydration. The zeta potential of fresh and lyophilized NPs was −26.16 ± 2.0 mV and − 21.79 ± 0.53 mV, respectively, indicating preserved surface charge characteristics. Importantly, the PDI remained low (0.18), suggesting maintained size uniformity. EE was highly consistent before and after lyophilization, at 76.56 ± 0.88% and 76.88 ± 1.25%, respectively (Fig. 2D). The minimal change in EE indicates effective retention of the drug within the polymeric matrix. Collectively, these findings demonstrate that the lyophilization process preserves both the structural integrity and therapeutic payload of the nanoparticles, supporting the stability of the formulation for use over a 4-week treatment period.

### 3.2 Cytotoxicity assessment of SN38-PLGA nanocomposites

In preclinical oncology study, it is standard practice to evaluate drug cytotoxicity in both malignant and non-malignant cell lines to determine therapeutic selectivity and potential off-target effects. In the present study, HCT116 cells were used as a model of human colorectal carcinoma, while HEK293T, a kidney-derived cell line, was included as a representative non-malignant control. Notably, in HEK293T cells, SN38-PLGA demonstrated significantly lower cytotoxicity compared with free SN38 (*p* < 0.001) (Fig. 2E). Similarly, in HCT116 cells, free SN38 treatment led to a marked reduction in cell viability compared with SN38-PLGA at concentrations above 40 nM (Fig. 2F). These findings indicate that encapsulation effectively mitigates SN38-induced cytotoxicity in both non-cancerous and cancerous cells. Moreover, the IC₅₀ values for SN38 and SN38-PLGA were higher in HEK293T cells than in HCT116 cells, confirming that both forms of SN38 exert greater cytotoxic effects on cancer cells than on non-cancerous cells.

### 3.3 SN38-PLGA nanocomposites induce DNA damage

Phosphorylated Chk1 (p-Chk1) serves as a marker of DNA damage–induced checkpoint activation, whereas γ-H2AX is a well-established indicator of DNA double-strand breaks and genomic DNA damage. To evaluate the dose-dependent DNA damage response induced by SN38-PLGA, HCT116 cells were treated with culture medium (negative control), free SN38 (10 ng/ml; positive control), or SN38-PLGA at concentrations of 5, 10, 20, and 40 ng/ml for 4 h. Compared with the negative control, both SN38 and SN38-PLGA treatments markedly increased p-Chk1 expression, with maximal activation observed at 20 ng/ml (Fig. 2G).

To further assess DNA double-strand break formation, γ-H2AX expression was examined by Western blotting, with vinculin used as a loading control. HCT116 cells were either left untreated or exposed to SN38 (20 nM) or SN38-PLGA (20 nM) for 24 h. Treatment with either free SN38 or SN38-PLGA markedly increased γ-H2AX expression, indicating that the SN38-loaded nanocomposites effectively induced DNA damage. Notably, γ-H2AX expression was higher in cells treated with free SN38 than in those treated with SN38-PLGA, consistent with cytotoxicity assay results demonstrating greater toxicity of free SN38 compared with SN38-PLGA (Fig. 2H).

### 3.4 SN38-PLGA nanoparticles maintain anti-tumor efficacy with reduced dosing frequency in a colitis induced cancer model

The AOM/DSS mouse model is a well-established experimental system for the study of CRC, particularly colitis-associated CRC. This model recapitulates key histopathological features of human disease, including the adenoma–carcinoma sequence and chronic inflammatory alterations, as well as characteristic molecular changes such as β-catenin activation and frequent dysregulation of the PI3K–Akt signaling pathway. These features render the AOM/DSS model a clinically relevant platform for evaluating chemotherapeutic efficacy (De Robertis et al., 2011; Pan et al., 2017). Accordingly, in the present study, we utilized the AOM/DSS model to assess the antitumor activity of SN38-PLGA.

**Fig. 3:**
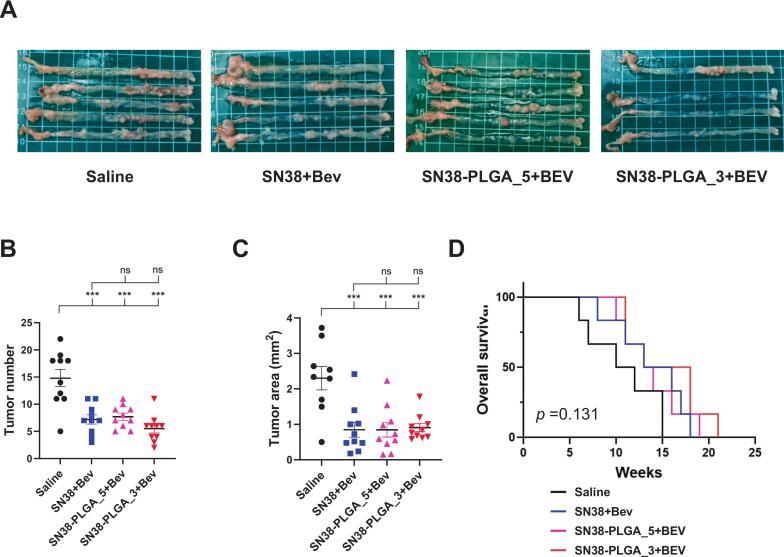
(A) Gross images of colon tumors in the AOM/DSS induced colorectal cancer model following treatment with SN38 or SN38-PLGA combined with bevacizumab. (B) Tumor counts illustrating the antitumor efficacy of SN38 or SN38-PLGA when combined with bevacizumab. (C) Quantification of tumor area per tumor demonstrating the antitumor effects of SN38 or SN38-PLGA in combination with bevacizumab. Data were analyzed using one-way ANOVA. (D) Kaplan–Meier survival curves showing overall survival of mice treated with saline, SN38 or SN38-PLGA combined with bevacizumab treated mice. Each group included six mice. Statistical significance: ns: non-significant. * *p* < 0.05, **p < 0.01 and ***p < 0.001.

After induction of colorectal cancer using AOM/DSS, mice were randomly assigned to four treatment groups: (1) saline, (2) SN38 plus bevacizumab, (3) SN38-PLGA administered five times per week plus bevacizumab, and (4) SN38-PLGA administered three times per week plus bevacizumab (Fig. 1). The gross morphology of the colon is shown in Fig. 3A. The mean tumor counts in these groups were 14.8, 7.2, 7.7, and 5.5, respectively (Fig. 3B). Administration of either free SN38 or SN38-PLGA significantly reduced tumor burden compared with saline (*p* < 0.001). There was no statistical difference between SN38 and SN38-PLGA (five times per week) (7.2 vs. 7.7, *p* = 0.987). Notably, reducing the SN38-PLGA dosing frequency from five to three injections per week maintained comparable antitumor efficacy (7.7 vs. 5.5, *p* = 0.455, one-way ANOVA).

The corresponding mean tumor areas were 2.3, 0.85, 0.85, and 0.91 mm^2^, respectively (Fig. 3C). Similarly, there was no significant difference between SN38 and SN38-PLGA administered five times per week (0.85 vs. 0.85, *p* = 1.000). Reducing the SN38-PLGA dosing frequency from five to three injections per week also preserved comparable efficacy (0.85 vs. 0.91, *p* = 0.997, one-way ANOVA). Both SN38 and SN38-PLGA treatments significantly reduced tumor burden compared with the saline. Moreover, decreasing the SN38-PLGA dosing frequency did not compromise its therapeutic effect, demonstrating that controlled-release nanoparticle delivery can achieve equivalent tumor suppression with reduced treatment intensity. We also evaluated mortality using Kaplan–Meier analysis, which demonstrated no significant differences among groups (*p* = 0.1307), likely reflecting the limited sample size (*n* = 6 per group). However, the log-rank test for trend was significant (*p* = 0.0357), indicating a stepwise survival benefit across treatments. Mice treated with SN38-PLGA exhibited longer survival than those receiving free SN38, whereas the saline group had the shortest survival (Fig. 3D).

### 3.5 PLGA-encapsulated SN38 alleviates colitis severity and preserves body weight

**Fig. 4:**
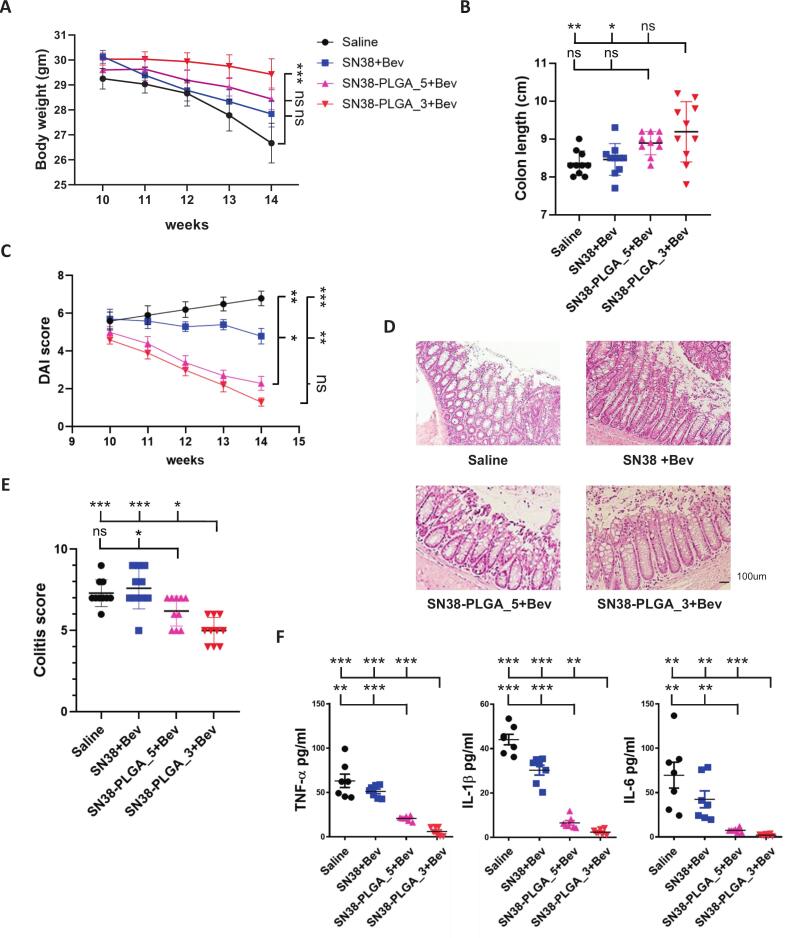
(A) Average body weight of mice treated with saline, SN38 (administered five times per week) with bevacizumab, SN38-PLGA_5 (administered five times per week) with bevacizumab, or SN38-PLGA_3 (administered three times per week) with bevacizumab. (B) Average colon length of each groups. (C) Disease activity index (DAI). (D) H&E staining of colon tissue. (E) Colitis scores. Scale bar: 100 μm. (F) Serum levels of TNF-α, IL-1β, and IL-6 in mice treated with saline, SN38, SN38-PLGA_5, or SN38-PLGA_3 combined with bevacizumab. Data were analyzed using one-way ANOVA. Statistical significance: ns: non-significant, * p < 0.05, **p < 0.01 and ***p < 0.001.

Irinotecan is known to cause both acute and delayed diarrhea, often resulting in weight loss (Yue et al., 2021), while cancer progression itself can lead to cachexia. In this study, mice treated with SN38 encapsulated in PLGA nanoparticles maintained a higher average body weight compared to those receiving either saline or free SN38 (Fig. 4A). Mean body weights were 26.7 g (saline), 27.8 g (SN38), 28.4 g (SN38-PLGA, five times per week), and 29.4 g (SN38-PLGA, three times per week), with a significant overall difference (*p* = 0.033). In addition, colon lengths were longer in the SN38-PLGA groups, averaging 8.4 cm (saline), 8.5 cm (SN38), 8.9 cm (SN38-PLGA, five times per week), and 9.2 cm (SN38-PLGA, three times per week) (*p* = 0.002; Fig. 4B).

The DAI was markedly lower in the SN38-PLGA groups than in the saline or SN38 groups, with scores of 6.8 (saline), 4.8 (SN38), 2.3 (SN38-PLGA, five times per week), and 1.3 (SN38-PLGA, three times per week) (*p* < 0.001; Figs. 4C). Consistently, histological colitis scores followed a similar trend, with values of 7.3 (saline), 7.6 (SN38), 6.2 (SN38-PLGA, five times per week), and 5.0 (SN38-PLGA, three times per week) (*p* < 0.001; Fig. 4D and E). Notably, SN38-PLGA administered five times per week significantly reduced colitis severity compared with free SN38 (6.2 vs. 7.6; *p* = 0.014). The SN38-PLGA group treated three times per week exhibited the lowest colitis score among all groups, which was also significantly lower than that of the five-times-per-week group (5.0 vs. 6.2; *p* = 0.043). Serum inflammatory cytokines, including TNF-α, IL-1β, and IL-6, were also markedly reduced in the SN38–PLGA treated groups, with the lowest levels observed in the three-times-per-week regimen, followed by the five-times-per-week regimen. In contrast, both the control and free SN38 groups exhibited higher cytokine levels (one-way ANOVA, p < 0.001 for TNF-α, IL-1β, and IL-6) (Fig. 4F).

These findings suggest that PLGA-encapsulated SN38 not only alleviates colon injury and inflammation but also improves body weight and overall disease severity compared to free SN38 or saline treatment.

### 3.6 Minimal risk of major organ damage from PLGA-encapsulated SN38

**Fig. 5:**
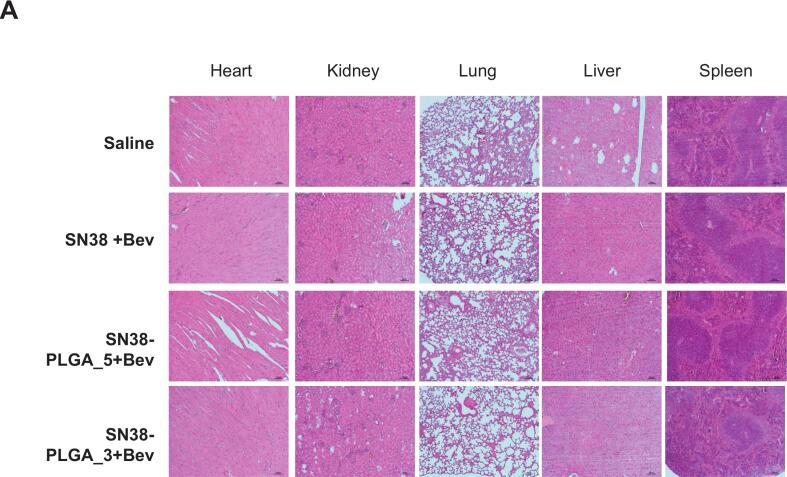
(A) Histological analysis of various mouse organs following treatment with saline, SN38 combined with bevacizumab, SN38-PLGA (administered five times per week) combined with bevacizumab, or SN38-PLGA (administered three times per week) combined with bevacizumab. Scale bar: 100 μm.

In the AOM/DSS-induced colitis-associated cancer mouse model, after treatment with saline, SN38, or SN38-PLGA, the mice were sacrificed to assess potential toxicity of the PLGA-encapsulated SN38 nanoparticles. Histological examination of major organs—including the heart, kidneys, lungs, liver, and spleen—revealed no pathological alterations, indicating that the nanocomposites exhibited no significant systemic toxicity. (Fig. 5).

## 4 Discussion

Our study demonstrated that SN38 encapsulated in PLGA nanoparticles exhibits comparable anti-tumor efficacy but with sustained effect as demonstrated by prolonging the treatment interval and reduced adverse effects compared to free SN38. PLGA is an ideal material for drug delivery, offering stabilized release properties, biocompatibility with tissues, and minimal systemic toxicity. (Sadat Tabatabaei Mirakabad et al., 2014) The clinical application of free SN38 is hindered by its poor aqueous solubility and rapid hydrolysis. Encapsulation of SN38 in PLGA provides layers of protection against degradation and ensures sustained drug release. While a number of studies have reported PLGA-based nanocomposites for SN38 delivery, the majority are limited to in vitro experiments or focus on antitumor efficacy in xenograft models of breast or glioblastoma cancers (Sepehri et al., 2014; Ebrahimnejad et al., 2009; Guo et al., 2013; Tseng et al., 2020; Yang et al., 2021). To our knowledge, this is the first study to evaluate the efficacy of SN38-encapsulated PLGA in treating colon cancer using an animal model.

In this study, mice treated with PLGA-encapsulated SN38 showed lower colitis scores, reduced disease activity indices, and higher body weights compared with those receiving free SN38 or saline, indicating reduced intestinal toxicity and improved overall health—consistent with in vitro findings showing that SN38-PLGA exhibited lower cytotoxicity than free SN38 in both cancerous and non-cancerous cell lines. Among the treatment groups, mice receiving SN38-PLGA three times per week showed the lowest colitis scores, indicating that encapsulation of SN38 within PLGA nanocomposites effectively mitigated gastrointestinal side effects. This delivery approach enabled a reduction in dosage and injection frequency while maintaining potent antitumor efficacy.

Clinically, irinotecan is known to cause two distinct types of diarrhea. Early-onset diarrhea is dose-dependent and presents severely (Grade 3/4) in up to 10% of patients. In contrast, delayed-onset diarrhea can occur at any dose level, is much more common—affecting up to 87% of patients—and is also frequently severe (Grade 3/4) in 16–22% of cases (Yang et al., 2025). Although irinotecan is the clinically approved prodrug of SN38 and the standard agent used in practice, free SN38 was chosen as the comparator in this study to permit direct molar dose matching and to avoid variability arising from metabolic activation. The mechanism underlying irinotecan-induced colitis involves luminal accumulation and reactivation of SN38, sustained disruption of intestinal microflora, and continual mucosal damage. After systemic administration, SN38 and its glucuronide conjugate are primarily excreted via feces (63.7% of the dose). In the gut, bacterial β-glucuronidase enzymes reconvert SN38 glucuronide into active SN38, which damages the intestinal mucosa and causes diarrhea (Stringer et al., 2008). SN38-induced mucosal injury impairs water and electrolyte absorption, enhances intestinal secretion, and may lead to renal or cardiovascular complications due to intravascular volume depletion (Stein et al., 2010). These complications may necessitate dose reduction or discontinuation of irinotecan therapy and can pose life-threatening risks.

Encapsulation of SN38 in PLGA nanocomposites helped mitigate these adverse effects. By providing controlled drug release and improved biodistribution, SN38-PLGA may minimize intestinal exposure to free SN38 and reduce the risk of mucosal toxicity. Clinically, this could translate to better treatment tolerance, fewer dose-limiting gastrointestinal events, and improved quality of life for patients undergoing irinotecan-based chemotherapy. Moreover, the ability to maintain antitumor efficacy at lower dosing frequencies suggests potential for more convenient treatment schedules, enhancing patient compliance and overall therapeutic outcomes.

## 5 Conclusion

PLGA-encapsulated SN38 nanocomposites exhibited significant antitumor effects on both colorectal cancer cell line and a colitis induced CRC mouse model. Importantly, SN38-PLGA achieved comparable antitumor efficacy while allowing a prolonged dosing interval and demonstrating an improved safety profile. These findings suggest that PLGA-based nanoparticles represent a promising drug delivery strategy to enhance the tolerability and therapeutic application of SN38 in colon cancer treatment.

## Declaration of generative AI use

ChatGPT (version GPT-5) was used to improve the English quality of this manuscript.

## CRediT authorship contribution statement

**Meng-Tzu Weng:** Writing – review & editing, Writing – original draft, Visualization, Funding acquisition, Formal analysis. **Shin-Yun Liu:** Methodology, Investigation, Formal analysis, Data curation. **Chia-Yueh Hsiung:** Writing – original draft, Methodology, Investigation, Data curation. **Meng-Chuan Wu:** Validation, Investigation, Data curation. **Tsung-Ying Lee:** Methodology, Investigation. **Been-Ren Lin:** Writing – review & editing, Visualization, Funding acquisition. **Yunching Chen:** Writing – review & editing, Visualization, Conceptualization. **Shu-Chen Wei:** Writing – review & editing, Writing – original draft, Supervision, Conceptualization.

## Funding

This study was supported by the grants from 10.13039/501100006477National Taiwan University, 10.13039/501100005762National Taiwan University Hospital (111-UN0054 to MTW and BRL), and The Liver Disease Prevention and Treatment Research Foundation, Taiwan (to SCW and MTW).

## Declaration of competing interest

The authors declare the following financial interests/personal relationships which may be considered as potential competing interests:

Meng-Tzu Weng, Been-Ren Lin reports financial support was provided by National Taiwan University, National Taiwan University Hospital. Meng-Tzu Weng, Shu-Chen Wei reports financial support was provided by The Liver Disease Prevention and Treatment Research Foundation, Taiwan. If there are other authors, they declare that they have no known competing financial interests or personal relationships that could have appeared to influence the work reported in this paper.

## Data Availability

Data will be made available on request.
